# Analysis of volatile organic compounds in ten types of tribute Rice based on headspace-gas chromatography-ion mobility spectrometry technology

**DOI:** 10.1016/j.fochx.2025.102520

**Published:** 2025-05-02

**Authors:** Qian-qian Jia, Yan-rong Ma, Wen-lu Bi, Ding-ding Su

**Affiliations:** Peking University Institute of Advanced Agricultural Sciences, Shandong Laboratory of Advanced Agricultural Sciences in Weifang, Shandong 261325, China

**Keywords:** Tribute Rice, Volatile organic compounds, HS-GC-IMS

## Abstract

Tribute rice, recognized for its unique quality and aroma, served as an imperial staple in ancient China. Many varieties of tribute rice are still preserved after hundreds of years. What makes them premium rice and why did they gain royal preference? What are their unique flavor characteristics? Little research has explored these aspects so far. This study highlights why tribute rice is favored by royalty and provides scientific insights into its distinct aromas. Through HS-GC-IMS analysis of ten representative tribute rice varieties, 74 volatile organic compounds were detected, including 69 characterized compounds: 21 aldehydes, 20 alcohols, 8 esters, 7 ketones, 4 heterocyclic compounds, 3 terpenoids, 2 acids, and 9 unclassified substances. Aldehydes and alcohols dominated the volatile profile, collectively accounting for over 50 % of the total relative content. These identified volatile components in ten tribute rice obtained by GC-IMS could be well distinguished by principal components. Meanwhile, orthogonal partial least squares-discriminant analysis (OPLS-DA) identified 15 key discriminatory volatiles. Notably, 2-acetyl-1-pyrroline (2-AP), known for its popcorn-like aroma, was detected across all samples—likely a trait that contributed to its selection as a royal offering. These findings advance our understanding of volatile compound variations in tribute rice across geographic origins, providing a scientific basis for origin authentication and quality enhancement.

## Highlights

• VOCs in 10 tribute rice varieties were accurately detected using HS-GC-IMS
• Identified a total of 74 peaks of tribute rice and 69 compounds
• Aldehydes and alcohols dominated (>50 % total content) the volatile profile.
• OPLS-DA identified 15 key markers enabling precise geographical origin discrimination.
• These findings provide a scientific basis for tribute rice origin authentication and quality enhancement.

## Graphical abstract

**Figure:**
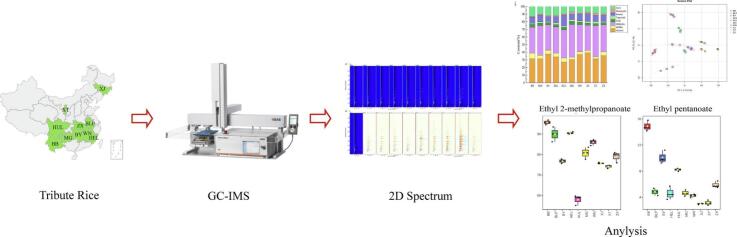

## 1 Introduction

Flavor is central to food enjoyment. Rice (*Oryza sativa L.*), a staple for over half the global population (Seck et al., 2012), stands as one of the world's three most crucial food crops. In 2021, rice production and consumption represented approximately 18.6 % and 18.7 % of the global staple output and intake, with rice contributing 81.3 % of the total (Food and Agriculture Organization of the United Nations, 2024). Rising living standards have elevated the demand for high-quality rice, positioning flavor as a primary consumer priority (Wang et al., 2020). Aromatic rice, in particular, has seen increased consumer preference and commands a premium in the market due to its distinct aroma and flavor, enjoying long-standing popularity across Asia. Consequently, assessing and researching rice aroma quality is essential for enhancing market competitiveness and advancing the agricultural economy (Addison et al., 2020; Hu et al., 2020). Thus, employing advanced analytical methods to identify and characterize the volatile organic compounds (VOCs) in rice is critical.

Regarding rice's volatile composition, typical volatile compounds include alkanes, aldehydes, ketones, esters, acids, alcohols, and heterocyclic compounds (Hu et al., 2020; Wang et al., 2021). Recent studies have analyzed rice flavor in various types, identifying key compounds that distinguish aromatic profiles. For instance, studies on Basmati, Jasmine, and other rice varieties identified thirteen primary volatiles responsible for aroma differentiation (Yang et al., 2008). A total of 93 aroma compounds were detected across aromatic and non-aromatic rice varieties, with sixteen compounds unique to aromatic types, including 5-methyl-3-heptan-2-one exclusively identified in Jasmine 85 and Cocodrie varieties (Bryant & Mcclung, 2010). Geographical discrimination of rice samples from India, China, and Vietnam successfully identified potential volatile biomarkers through GC–MS-based metabolomic profiling combined with multivariate analysis (Ch et al., 2021). Headspace-Gas chromatography-ion mobility spectrometry (HS-GC-IMS) was employed to analyze 53 fragrant rice samples, enabling successful varietal differentiation through chemometric analysis and digital imaging (Chen et al., 2022). Additionally, key variables influencing the sensory quality of Chinese rice flavor were screened using samples from nine representative cultivation regions (Zhou et al., 2023). However, despite considerable research on rice flavor, the study of tribute rice remains underexplored.

Historically cultivated exclusively for royalty, tribute rice is renowned for its superior quality and consumer recognition (Jiang et al., 2013). Cultivated in regions with optimal ecological conditions, tribute rice features a crystalline appearance, delicate texture, and rich nutritional content. For example, Wannian tribute rice, a late-maturing indica variety, has been cultivated as a tribute since the Northern and Southern Dynasties (Zeng, 2021). Similar in morphology to wild rice, this variety has slender, shuttle-shaped grains and a pure white texture. It thrives in mineral-rich environments with unique microclimates—high altitudes, deep ridges, distinctive sunlight, and variable spring temperatures—contributing to limited but superior yields (Chen, 2017). Rice is cultivated across tropical and northern temperate zones, with photoperiod and temperature variations driving its extensive genetic diversity (Reig-Valiente et al., 2016; Zhang et al., 2021). In high-latitude regions such as Liaoning, prolonged sunshine duration and significant diurnal temperature fluctuations promote amylose and protein accumulation while reducing chalkiness, thereby enhancing milling yield and cooking quality (Li et al., 2018). Conversely, low-latitude areas like Guangdong experience high temperatures and humidity, which elevate chalkiness rates, diminish milling efficiency, and inhibit nocturnal amylose synthesis—factors that collectively compromise sensory quality (Li et al., 2018). These latitudinal differences underscore distinct varietal adaptability in rice (Jin et al., 2013). Furthermore, latitude-dependent solar radiation modulates the reproductive phase duration, indirectly influencing grain filling dynamics and subsequently shaping grain morphology and nutritional profiles (Li et al., 2018; Reig-Valiente et al., 2016). Rice is widely cultivated across China, with Tribute Rice standing out as a premium variety highly prized by consumers for its exceptional grain quality and culturally significant historical legacy (Jiang et al., 2013). Currently, tribute rice retains ancestral phenotypes and genetic integrity due to region-specific cultivation and limited yields. However, it faces critical challenges, including stagnant genetic improvement, varietal degradation, climate vulnerability, and incompatibility with mechanized farming. Some varieties now risk extinction, underscoring the urgency of research on genetic conservation, genomic profiling, and flavor chemistry in tribute rice.

To investigate the flavor characteristics of tribute rice across diverse regions, this study selected representative varieties cultivated in major agricultural zones spanning low to high latitudes. The volatile compounds of these tribute rice samples were analyzed using HS-GC-IMS, integrated with multivariate pattern recognition analysis to identify potential volatile markers associated with their geographical origins. This research aims to establish a standardized evaluation framework for premium tribute rice and provide a scientific basis for origin authentication. These findings advance the understanding of region-specific flavor profiles in tribute rice and support the conservation of these heritage cultivars.

## 2 Materials and methods

### 2.1 Materials

**Fig. 1:**
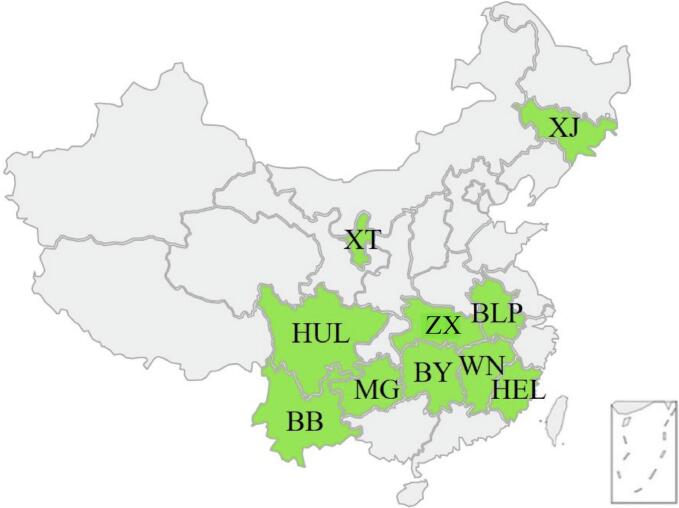
Specific sampling provinces of ten tribute rice.

Ten types of harvested tribute rice were selected as test materials, with all samples commercially available and sourced from ten different provinces across China. The selected varieties include: Babao Tribute Rice (BB, Babao Town, Guangnan County, Yunnan Province), Bailianpo Tribute Rice (BLP, Huaiyuan County, Anhui Province), Baiyun Tribute Rice (BY, Xiangxi Tujia and Miao Autonomous Prefecture, Hunan Province), Hailong Tribute Rice (HEL, Hailong Township, Ninghua County, Fujian Province), Huanglong Tribute Rice (HUL, Huanglong Township, Yuechi County, Sichuan Province), Mao Tribute Rice (MG, Meitan County, Guizhou Province), Wannian Tribute Rice (WN, Wannian County, Shangrao City, Jiangxi Province), Xi Jiang Tribute Rice (XJ, Xi Jiang Town, Tonghua County, Jilin Province), Xingtang Tribute Rice (XT, Lingwu City, Ningxia Hui Autonomous Region), and Zhuxi Tribute Rice (ZX, Zhuxi County, Hubei Province). The specific geographical locations of each sample are illustrated in Fig. 1.

### 2.2 Flavor analysis

Flavor determination of the rice samples was conducted using HS-GC-IMS, following a slightly modified version of the method developed by Chen et al. (2022). The analysis was conducted using a FlavourSpec® GC–IMS system (G.A.S., Dortmund, Germany) equipped with a PAL RSI autosampler unit (CTC Analytics AG, Zwingen, Switzerland). Specifically, 5 g of rice sample was weighed into a 20 mL headspace vial and incubated at 80 °C for 30 min prior to injection. The HS-GC-IMS analysis conditions were as follows: the column used was MXT-WAX (0.53 mm × 15 m, 1 μm); nitrogen served as both the carrier gas (EPC2) and drift gas (EPC1); the injection volume was 500 μL, with an injection temperature of 65 °C and an IMS temperature of 40 °C. The initial flow rates for EPC1 and EPC2 were set to 75 mL/min and 2 mL/min, respectively, gradually increasing to 100 mL/min within 30 min to achieve optimal separation. Each analysis ran for a total of 30 min, and each rice sample was tested in triplicate.

HS-GC-IMS data processing was carried out using the instrument's proprietary software, which includes the VOCal, Reporter, and Gallery Plot plug-ins. VOCal was used for spectral analysis and compound identification, leveraging the NIST and IMS databases for qualitative analysis. The Reporter plug-in enabled direct comparisons of spectral differences between samples, while the Gallery Plot plug-in produced fingerprints, facilitating both visual and quantitative comparisons of the volatile compounds across samples.

### 2.3 Relative odor average value

The relative contribution of each component to the volatile flavor profile of tribute rice was evaluated using the Relative Odor Average Value (ROAV), based on the relative content and threshold values of the compounds. This evaluation method was adapted from Li et al. (2024). HS-GC-IMS identified the VOC profiles in aromatic and non-aromatic rice cultivars. Chemometrics differentiated the two rice categories and identified key VOCs. Artificial sensory evaluation determined the aroma intensity of rice varieties.The correlation between sensory evaluation and VOCs in rice cultivars was explored. Xiong et al. (2024) conducted a comparative study using HS-GC-IMS to analyze volatile compound profiles in aromatic and non-aromatic rice cultivars and their correlation with sensory evaluation. The component with the highest contribution to the overall flavor of the sample was assigned an ROAV of 100. The ROAV for other volatiles was calculated as follows:

$$\text{ROAV}=\left(\frac{C_{i}}{C_{\max}}\times \frac{T_{\max}}{T_{i}}\right)\times 100$$

where C_i_ and T_i_ represent the relative mass fraction (%) and sensory threshold of each compound, respectively. C_max_ and T_max_ refer to the relative mass fraction (%) and sensory threshold, respectively of the compound that contributes the most to the overall flavor of the sample.

### 2.4 Experimental design and statistical analysis

The results of the Hierarchical Cluster Analysis (HCA) for various volatile organic compounds were visualized as heatmaps and dendrograms. Unsupervised Principal Component Analysis (PCA) and Orthogonal Partial Least Squares Discriminant Analysis (OPLS-DA) were conducted using SIMCA software (version 14.1) for comprehensive data analysis. Additionally, Variable Importance in Projection (VIP) scores were utilized to pinpoint volatiles that exhibited significant differences between groups, aiding in the identification of key distinguishing compounds.

## 3 Results

### 3.1 HS-GC-IMS spectral analysis of ten types of tribute Rice

**Fig. 2:**
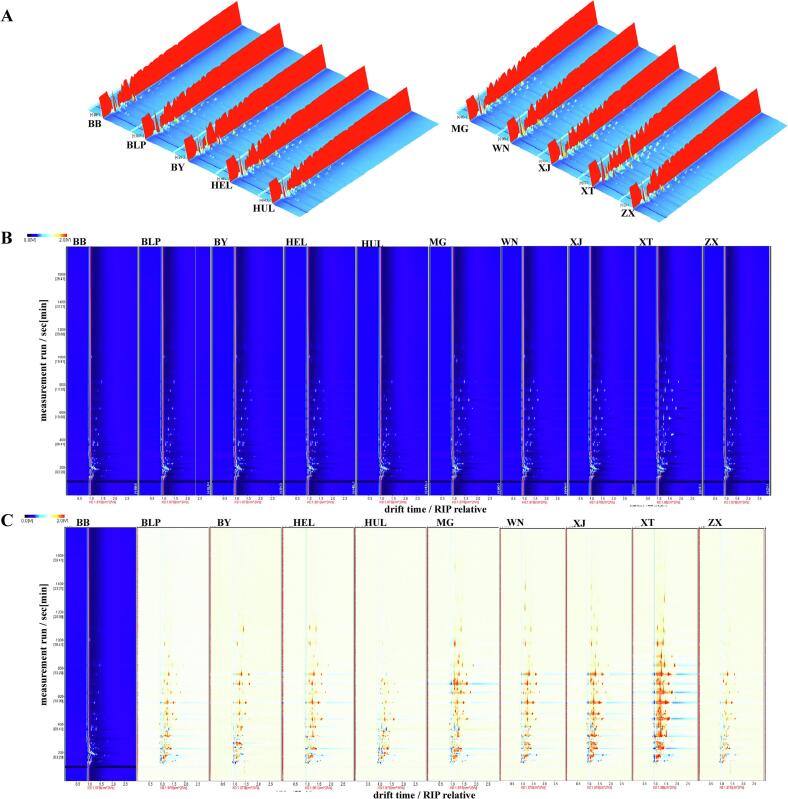
The 3D map (A), 2D map (B), and difference comparison map (C) of volatile flavor compounds in ten types of tribute rice.

Fig. 2A illustrates the three-dimensional HS-GC-IMS profiles of VOCs in the ten tribute rice samples, with the x-axis, y-axis, and z-axis representing ion mobility time, GC retention time, and signal peak intensity, respectively (Liu et al., 2022; Yang et al., 2021). These three-dimensional maps provide raw data on all compounds, allowing for precise identification of signal peak intensities and positions for each volatile flavor compound (Wang et al., 2020). Variations in volatile compounds across the tribute rice samples are visually represented in Fig. 2A.

To better distinguish the profiles of volatile compounds among the tribute rice samples, ion migration times and reaction peak positions were normalized and presented in a two-dimensional HS-GC-IMS plot (Fig. 2B). The plot background is blue, with a red vertical line at the horizontal coordinate of 1.0 indicating the Reactive Ion Peak (RIP) (Yang et al., 2021). The horizontal axis shows ion migration time, while the vertical axis shows HS-GC-IMS retention time. Each point near the RIP peak represents a volatile organic compound, with color intensity indicating concentration: white (lower), red (higher), and deeper red (highest) (Liu et al., 2022; Yang et al., 2021; Zhang et al., 2024). Volatile compounds appear within drift times of 1.0–12.0 ms and retention times of 100–1000 s (Zhang et al., 2024).

Preliminary GC-IMS analyses demonstrated that Babao Tribute Rice contained the lowest number of detectable VOCs among all samples (Fig. 2B). This simplified chemical baseline improves the statistical resolution for differentiating subtle variations in aroma profiles among other tribute rice varieties. For comparative analysis, we employed a differential comparison method using Babao Tribute Rice's spectrum as the reference, by subtracting the spectra of other samples from it. In this mode, white represents VOC levels comparable to the reference, red signifies higher concentrations, and blue denotes lower levels. As illustrated in Fig. 2C, when compared with Babao Tribute Rice, some samples exhibited increased VOC content while others showed decreased levels, particularly within the retention time range of 100–300 s.

### 3.2 Qualitative results and fingerprint analysis of volatile substances in ten types of tribute Rice samples

**Fig. 3:**
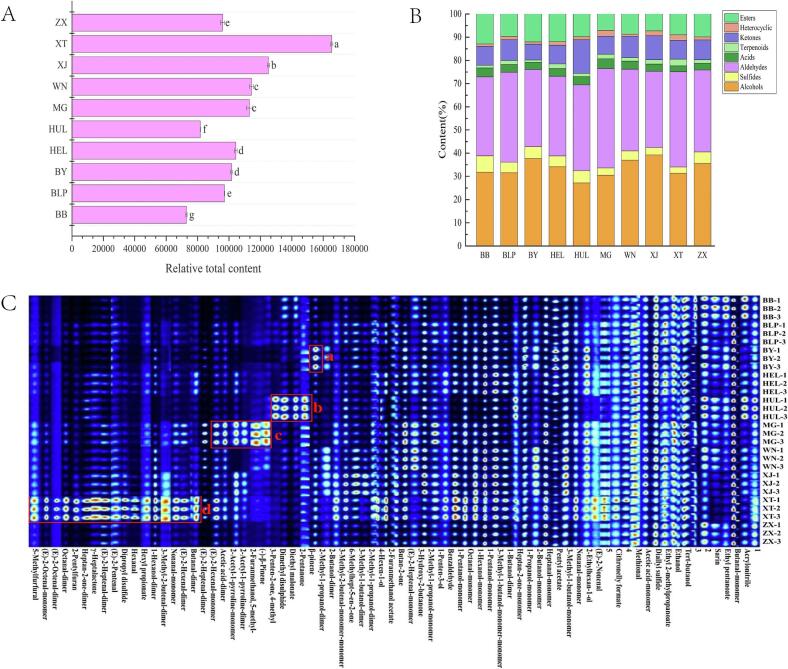
The relative total content of volatile compounds (A), the classification of various compounds (B) and fingerprinting plots (C) in ten types of tribute rice.

Volatile flavor compounds in all Tribute Rice samples were qualitatively analyzed using HS-GC-IMS. Detailed information on the identified volatile flavor compounds across all samples is provided in Electronic Exhibit 1. To facilitate a visual comparison of volatile compound differences among samples, fingerprint plots were generated (Fig. 3C), where rows represent different tribute rice samples, and columns represent detected volatile compounds. Among the 74 detected peaks, 69 known volatile compounds and 5 unidentified ones were identified, comprising 21 aldehydes, 20 alcohols, 8 esters, 7 ketones, 4 heterocyclic compounds, 3 terpenoids, 2 acids, and 9 unknown substances (Table S1). In these fingerprint plots, black indicates lower relative content, while red signifies higher relative content.

The relative total content of volatile compounds was highest in Xingtang Tribute Rice, followed by Xijiang Tribute Rice and Wannian Tribute Rice. In contrast, Babao Tribute Rice, Bailianpo Tribute Rice, Hailong Tribute Rice, and Zhuxi Tribute Rice exhibited relatively fewer volatile substances (Fig. 3). Fig. 3B shows that aldehydes (32.9–42.8%) were the most abundant compound class in all tribute rice samples, followed by alcohols (27.2–39.2 %), esters (7.0–12.8 %), ketones (6.8–14.5 %), sulfides (2.6–7.0 %), and acids (2.5–4.2 %). Conversely, terpenoids and heterocyclic compounds were present in lower amounts across all tribute rice samples. Aldehydes, alcohols, and esters were the most prevalent volatile compounds, aligning with previously reported volatile profiles in rice (Xiong et al., 2024). These compounds, with their low odor thresholds, are significant contributors to rice's distinctive flavor characteristics.

Aldehydes are recognized as the most important volatile components in raw rice and are mainly produced via lipid oxidation and decomposition (Zheng et al., 2024). These substances contribute fresh and fruity aromas to rice grains. In all tribute rice samples, substances such as butanal-M, nonanal-M, (E)-2-heptenal-M, (E)-2-heptenal-D, and octanal-M were found in relatively high amounts. Xingtang Tribute Rice contained the highest concentration of volatile compounds such as butyraldehyde-D, trans-2-hexenal-D, nonanal-D, 3-methyl-2-butenal-D, hexanal, (Z)-2-pentenal, heptanal-D, octanal-D, (E)-2-octenal-D, and (E)-2-octenal-M (Fig. 3C region d). Among them, hexanal is an important volatile compound in rice, which can serve as an indicator of lipid oxidation, mainly contributing to flavors such as fruity, grass, and green (Kasote et al., 2021).

Alcohols are considered secondary products from the oxidation of unsaturated fatty acids, arising from the further breakdown of aldehydes (Lina & Min, 2022). They represent the second most abundant group of volatiles in rice and are notably present in higher quantities in Baiyun Tribute Rice, Wannian Tribute Rice, and Xijiang Tribute Rice. The primary alcohols detected include 1-pentanol-M, 3-methyl-1-butanol-M, 1-propanol-M, ethanol, and 1-hexanol-M. Among these, hexanol imparts green, herbaceous, and sweet notes, while ethanol and 1-pentanol add ethereal, alcoholic, and fermented flavors.

Esters, generated through esterification reactions between alcohols and acyl-CoAs derived from both the fatty acid lipoxygenase pathway and amino acid metabolism (GonzaLez et al., 2009), were also prominent in tribute rice. Notable esters detected included γ-heptalactone, pentyl acetate, ethyl 2-methylpropanoate, 2-furanmethanol acetate, ethyl pentanoate, hexyl propionate, and diethyl malonate, which contribute significantly to the aroma during the cooking process (Maraval et al., 2008). The ester content was highest in Babao Tribute Rice, Baiyun Tribute Rice, and Hailong Tribute Rice (Fig. 3B).

Ketones, which are typically products of alcohol oxidation or ester decomposition, impart fatty and slightly burnt notes. A limited range of ketones was detected in tribute rice, including 6-methyl-5-hepten-2-one, heptan-2-one-D, butan-2-one, 2-pentanone, 3-hydroxy-2-butanone, and 4-methyl-3-penten-2-one. The highest relative content of ketones was found in Huanglong Tribute Rice, predominantly 2-pentanone and 4-methyl-3-penten-2-one (Fig. 3C, region b).

Heterocyclic compounds such as furans, pyrazines, thiophenes, thiazoles, pyrroles, imidazoles, and pyridines are major volatile compounds produced via Maillard reactions (Hu et al., 2020). Three key heterocyclic compounds were identified in the tribute rice samples: 5-methylfurfural, 2-pentylfuran, and 2-acetyl-1-pyrroline. In particular, 2-pentylfuran was detected in the highest concentration in Xingtang Tribute Rice, imparting a nutty aroma at low concentrations and a soybean-like odor at higher levels (Zeng et al., 2007).

2-Acetyl-1-pyrroline, a key compound in rice aroma, was found at the highest concentration in Mao Tribute Rice, followed by Xijiang and Xingtang Tribute Rice (Fig. 3C, region c). Known for its characteristic popcorn scent due to its low odor threshold, 2-AP is enzymatically synthesized during rice growth and is one of the primary aroma contributors in fragrant rice. The 2-AP molecule, notable for its pyrroline ring, is highly unstable, making it a distinctive and easily perceived aromatic compound in rice (Hu et al., 2020; Kasote et al., 2021; Prodhan, 2020).

### 3.3 Multivariate statistical analysis

**Fig. 4:**
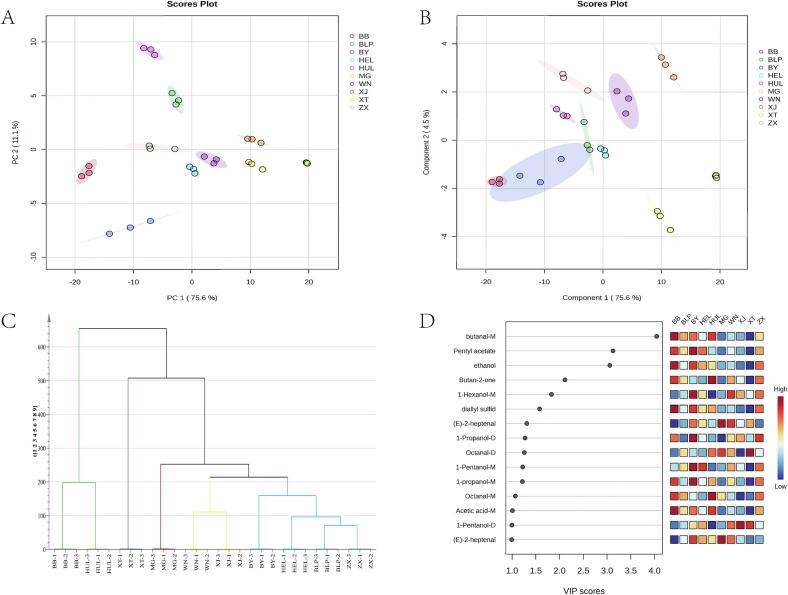
Principal component analysis (A), partial least squares-discriminant analysis (B), hierarchical clustering (C) and and VIP Plot of Potential Volatile Compounds (D) in ten types of tribute rice.

The unsupervised PCA model based on HS-GC-IMS data revealed distinct clustering characteristics among all rice varieties. Fig. 4A shows that PC1 and PC2 explain 75.6 % and 11.1 % of the variance, respectively, capturing a total of 86.7 % of the dataset's variance. This combined contribution, exceeding 80 %, effectively captures the primary variation within the dataset. In the figure, tribute rice samples are distributed across four quadrants: Xijiang Tribute Rice is located in the first quadrant, Baiyun Tribute Rice and Babao Tribute Rice in the third quadrant, Huanglong Tribute Rice and Bailianpo Tribute Rice in the second quadrant, and Wannian Tribute Rice, Mao Tribute Rice, and Xingtang Tribute Rice in the fourth quadrant. This distribution suggests considerable diversity in the volatile compound compositions of the different tribute rice samples.

To directly illustrate inter- and intra-group metabolic differences, a cluster analysis was conducted on the study data (Fig. 4C). Samples within each group clustered closely, showing clear differentiation from other groups, which reflects low variability within groups and significant differences between groups. Notably, Xingtang Tribute Rice exhibited the most distinct profile compared to other samples, while Baiyun, Hailong, Bailianpo, and Zhuxi Tribute Rice shared some compositional similarities. These findings indicate that each of the ten tribute rice varieties possesses unique metabolic characteristics, as demonstrated by multivariate statistical analysis.

The application of supervised PLS—DA enhanced the separation of tribute rice varieties from different regions and aided in identifying volatile markers that distinguish the ten types of tribute rice. The PLS—DA score plot (Fig. 4B) highlights differences between categories along the horizontal axis, representing the component scores as predicted by the model. Model validation via leave-one-out cross-validation yielded a goodness of fit (R^2^Y = 0.98) and a goodness of prediction (Q_2_ = 0.96), both exceeding 0.95, indicating high accuracy and robustness. To mitigate potential overfitting in supervised classification, 100 permutation tests were conducted to validate the model's reliability further.

Variable Importance in Projection (VIP) values for the volatile compounds were derived from the PLS—DA model, identifying 10 compounds with VIP values greater than 1, as listed in Fig. 4D. Of these, 15 substances had VIP values exceeding 1, with butanal-M, pentyl acetate, ethanol, butan-2-one, and 1-hexanol-M showing particularly high values. These findings align with prior studies that used GC-IMS to analyze rice volatiles. For instance, Xiong et al. (2024) used GC-IMS to explore flavor distinctions between aromatic and non-aromatic rice, identifying a positive correlation between 1-pentanol, 2-acetyl-1-pyrroline-D, and 1-hexanol-M with sensory flavor. Similarly, Chen et al. (2022) employed GC-IMS to examine VOC differences between Guangxi and Wuchang aromatic rice, finding that 2-butanone, 2-heptanone, pinene, heptanal, 2-methyl-1-butanol acetate, and octanal were key flavor components.

### 3.4 Comparison of major volatile flavor substances in ten types of tribute Rice

**Fig. 5:**
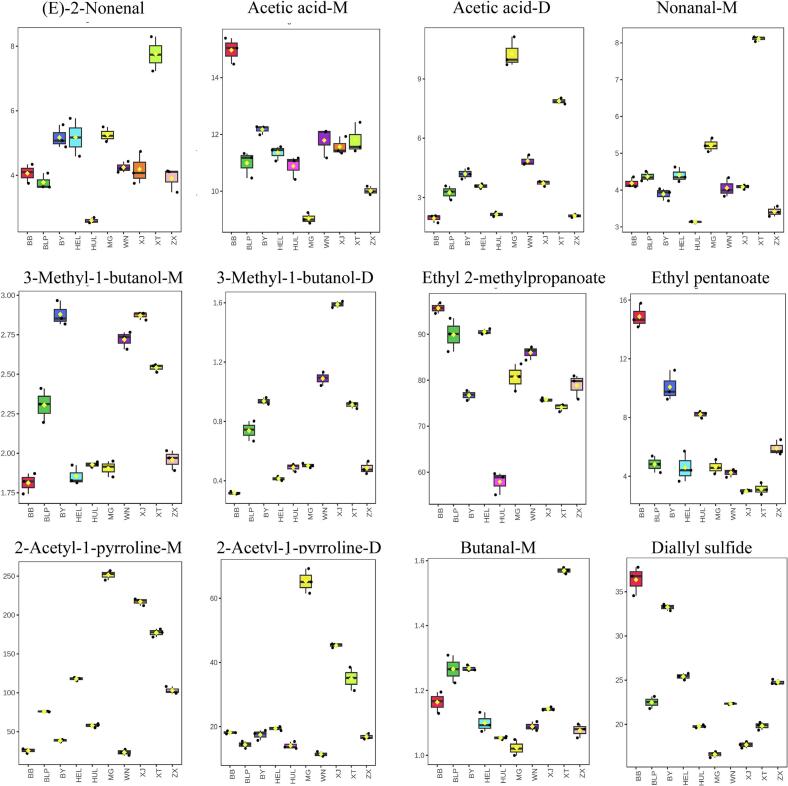
Volatile compounds in tribute rice with ROAV values.

The contribution of a volatile flavor component to the overall aroma profile depends on both its odor threshold and its relative concentration. The Relative Odor Activity Value (ROAV) is widely used to assess the impact of volatile flavor compounds on overall flavor (Hu et al., 2020). In this study, volatile compounds with ROAV values greater than 1 in the tribute rice samples were identified as primary contributors to the flavor (Fig. 5, Table S2). Common primary volatiles in the twelve samples included acetic acid-M and acetic acid-D (vinegar, acidic), diallyl sulfide (sulfurous), 3-methyl-1-butanol-M (pungent), butanal-M (chocolate), 3-methyl-1-butanol-D (pungent), (E)-2-nonenal, nonanal-M (fatty, citrus-like), ethyl-2-methylpropanoate (fruity), ethyl pentanoate (fruity, apple), and both 2-acetyl-1-pyrrolidine-M and 2-acetyl-1-pyrrolidine-D (popcorn). These compounds primarily contributed pungent, fatty, fruity, and popcorn-like flavors.

The volatile compounds (E)-2-nonenal, nonanal-M, 3-methyl-1-butanol-D, and butanal were most abundant in Xingtang Tribute Rice, while acetic acid-M, ethyl pentanoate, and diallyl sulfide were predominant in Babao Tribute Rice. In Mao Tribute Rice, the highest concentrations were observed for acetic acid-D, 2-acetyl-1-pyrrolidine-M, and 2-acetyl-1-pyrrolidine-D. Conversely, ethyl-2-methyl propanoate was found in relatively low concentrations in Huanglong Tribute Rice.

## 4 Discussion

Aroma quality is a critical attribute that enhances the commercial value of rice in global markets (Hu et al., 2020). As a complex biochemical system, rice aroma arises from the synergistic effects of multiple volatile compounds rather than isolated components. Over 500 volatile compounds have been identified across aromatic and non-aromatic rice varieties (Verma & Srivastav, 2018). In this study, we employed GC-IMS to analyze volatile compounds in ten tribute rice varieties, identifying 74 compounds, including 69 characterized volatiles and 5 unknown substances. The detected compounds comprised 21 aldehydes, 20 alcohols, 8 esters, 7 ketones, 4 heterocyclic compounds, 3 terpenes, 2 acids, and 9 unclassified substances. Aldehydes and alcohols accounted for 55 % of the total volatile composition, while ketones, esters, and acids were also present in substantial amounts. This study is in agreement with the results of studies using GC-IMS for aromatic, glutinous, and nonaromatic rice (Chen et al., 2023). Previous studies utilizing gas chromatography–mass spectrometry (GC–MS) have detected 198 volatile compounds in Chinese japonica rice, with ketones, alcohols, and aldehydes playing pivotal roles in shaping its raw grain volatile profile (Zhao et al., 2022). Additionally, substantial quantities of alkanes were identified, constituting 50 %–60 % of total volatile components in Chinese japonica rice. The absence of alkanes in our study primarily stems from methodological differences between GC-IMS and GC–MS. Alkanes are not important contributors to aroma characteristic for rice due to their relatively high threshold values (Zhao et al., 2022). Compared to GC–MS, GC-IMS offers superior separation efficiency and sensitivity (detection limits at parts per trillion by volume, pptv), while maintaining high mass and time resolution (Parastar & Weller, 2024). GC-IMS offers significant advantages for real-time sample analysis without requiring extensive pretreatment, rendering it particularly suitable for rapid screening applications (Li et al., 2024). However, its reliance on direct headspace injection enhances sensitivity to low-boiling-point compounds (C_2_-C_10_), which can make the detection of volatile benzene derivatives and nitrogen-containing compounds with higher molecular weights or boiling points challenging (Li et al., 2022).

Previous research highlights that aldehydes and alcohols are significant contributors to aroma due to their pleasant scents and low odor thresholds (Dajanta et al., 2011). Ketones, such as 3-hydroxy-2-butanone, 2-pentanone, and 2-heptanone, also impact flavor due to their low thresholds and distinct aromas (Api et al., 2020). Esters, including pentyl acetate, isobutyric acid ethyl ester, pentanoic acid ethyl ester, and ethyl valerate, contribute floral and fruity notes, enhancing the rice's appealing aroma profile. Based on these findings, tribute rice exhibits a range of floral, fruity, and popcorn-like aromas, which may explain its historical status as a premium rice preferred by royalty. A key compound, 2-acetyl-1-pyrroline, recognized for its popcorn-like scent, was detected in all ten tribute rice types with a ROAV above 1, indicating its significant role in the aromatic profile of tribute rice. This supports the inference that breeding fragrant rice varieties may have historical ties to tribute rice. ROAV analysis identified key volatiles, including 3-methyl-1-butanol, butanal, (E)-2-nonenal, methyl-2-methylpropanoate, 2-acetyl-1-pyrroline, ethyl pentanoate, and nonanal, highlighting the flavor diversity among tribute rice varieties.

Results indicate that the ten Tribute Rice varieties exhibit distinct profiles influenced by variations in compound type and content. These flavor variations are influenced not only by genetic factors but also by climatic conditions, soil composition, environmental parameters, and cultivation practices(Behera & Panda, 2023; Ch et al., 2021; Y. Li & Tao, 2023). For example, Wannian Tribute Rice grows in nutrient-rich mountain ridges nourished year-round by spring water containing decayed organic matter and minerals (Zeng Y., 2021). Babao Tribute Rice, grown at a lower latitude, benefits from more than 6 h of sunlight, while Baiyun Tribute Rice is cultivated in mountainous areas with significant diurnal temperature changes and a long frost-free period. Xingtang Tribute Rice, planted at a higher latitude, benefits from extended daylight, and Xijiang Tribute Rice grows at high altitudes (30–1200 m above sea level), where monsoonal influences create a unique microclimate. Latitudinal variation-induced solar radiation modulates grain morphology and nutritional quality by regulating the duration of the reproductive phase (Jin et al., 2013). Therefore, the volatile profiles of tribute rice samples are closely linked to their geographical environments.

Identifying the geographical origin of rice is essential for tracking production, preventing adulteration, and protecting brand value (Behera & Panda, 2023; Qian et al., 2019; Teye et al., 2019). VOCs have proven effective in distinguishing geographical origin, variety, and quality in food and agricultural products (Lim et al., 2018; Song et al., 2020). The potential of HS-GC-IMS as a tool for determining the geographical origin of rice was demonstrated by Chen et al. (2022). Similarly, He et al. employed GC-IMS to analyze volatile organic compounds in Ophiopogonis Radix from different regions, identifying 12 characteristic compounds that effectively discriminated samples from three production areas (He et al., 2022). Meanwhile, it was shown that the volatile compounds detected by GC-IMS combined with principal component analysis and cluster analysis could clearly distinguish between different colored foxtail millets (Jin et al., 2023). In this study, we analyzed volatile compounds in ten tribute rice varieties and constructed their fingerprint profiles. Fifteen differential volatile compounds were identified as markers for distinguishing the unique flavors of various tribute rice samples. These findings provide valuable insights for differentiating the geographical and varietal origins of tribute rice.

## 5 Conclusion

This study investigated the types and concentrations of volatile organic compounds in ten types of Tribute Rice samples. GC-IMS effectively identified volatile organic compounds, with aldehydes and alcohols as the predominant components. Orthogonal partial least squares-discriminant analysis (OPLS-DA) identified 15 key discriminatory volatile compounds that effectively distinguish the unique flavor profiles of different tribute rice samples. Among these, seven volatiles—acetic acid monomer, diallyl sulfide, ethyl 2-methylpropanoate, ethyl pentanoate, methional, 2-acetyl-1-pyrroline - monomer, and 2-acetyl-1-pyrroline dimer—were highlighted as essential contributors to flavor characteristics. These markers not only reflect geographical specificity but also correlate with sensory attributes historically associated with royal preferences. In this study, based on GC-IMS technology, the fingerprint of volatile compounds in ten tribute rice varieties was established. This work provides a reference framework for enhancing tribute rice quality and flavor profiles. Furthermore, it delivers valuable insights for geographical indication protection, varietal improvement, and novel product development. The study analyzed ten rice varieties with restricted sample sizes per geographical region. While GC-IMS offers high sensitivity, its reliance on relative abundance reporting rather than absolute concentration measurement limits cross-study comparability. Future work could improve detection accuracy by integrating hyperspectral imaging or comprehensive two-dimensional gas chromatography coupled with time-of-flight mass spectrometry (GC × GC-TOF-MS). Additionally, consumer preference trials correlating volatile organic compound profiles with hedonic scores would bridge chemical data and sensory evaluation.

## CRediT authorship contribution statement

**Qian-qian Jia:** Writing – review & editing, Writing – original draft, Formal analysis, Data curation. **Yan-rong Ma:** Writing – original draft, Formal analysis, Data curation. **Wen-lu Bi:** Writing – review & editing, Validation, Supervision. **Ding-ding Su:** Writing – review & editing, Visualization, Validation, Project administration, Funding acquisition.

## Funding information

This study was supported by the Taishan Industrial Experts Program and the Yuandu Industry Leading Talents Project.

## Declaration of competing interest

The authors declare that they have no known competing financial interests or personal relationships that could have appeared to influence the work reported in this paper.The authors declare that they have no known competing financial interests or personal relationships that could have appeared to influence the work reported in this paper.

## Data Availability

Data will be made available on request.

## Appendix A Supplementary data

Supplementary material Image 1
