# Supplementary material for: Analysis of volatile organic compounds in ten types of tribute Rice based on headspace-gas chromatography-ion mobility spectrometry technology
Source: Food Chem X. 2025 May 2;28:102520. doi: 10.1016/j.fochx.2025.102520 (PMC12139503; doi:10.1016/j.fochx.2025.102520)
Supplement: Supplementary file 1 — Supplementary material [file mmc1.docx]

Table S1 The information of VCs in ten tax rice varieties identified by GC-IMS.

| Number | Volatile compound | CAS# | Formula | MW | | RI | Rt [sec] | Dt [a.u.] | Comment |
| --- | --- | --- | --- | --- | --- | --- | --- | --- | --- |
| 1 | 5-Methylfurfural | 620-02-0 | C6H6O2 | 110.1 | | 1576.4 | 1296.674 | 1.47362 |  |
| 2 | Benzaldehyde | 100-52-7 | C7H6O | 106.1 | | 1509.8 | 1091.484 | 1.15123 |  |
| 3 | (E)-2-Nonenal | 18829-56-6 | C9H16O | 140.2 | | 1528.8 | 1146.386 | 1.4091 |  |
| 4 | 2-Ethylhexan-1-ol | 104-76-7 | C8H18O | 130.2 | | 1504.2 | 1075.853 | 1.41612 |  |
| 5 | Acetic acid | 64-19-7 | C2H4O2 | 60.1 | | 1477.8 | 1004.755 | 1.05583 | Monomer |
| 6 | Acetic acid | 64-19-7 | C2H4O2 | 60.1 | | 1465.6 | 973.611 | 1.16023 | Dimer |
| 7 | (E)-2-Octenal | 2548-87-0 | C8H14O | 126.2 | | 1429.4 | 886.795 | 1.33031 | Monomer |
| 8 | (E)-2-Octenal | 2548-87-0 | C8H14O | 126.2 | | 1429.1 | 885.935 | 1.81432 | Dimer |
| 9 | Nonanal | 124-19-6 | C9H18O | 142.2 | | 1400.6 | 823.064 | 1.47831 | Monomer |
| 10 | Nonanal | 124-19-6 | C9H18O | 142.2 | | 1399.9 | 821.536 | 1.93257 | Dimer |
| 11 | 6-Methylhept-5-en-2-one | 110-93-0 | C8H14O | 126.2 | | 1348.6 | 719.592 | 1.17387 |  |
| 12 | (E)-2-Heptenal | 18829-55-5 | C7H12O | 112.2 | | 1332.9 | 691.035 | 1.25271 | Monomer |
| 13 | (E)-2-Heptenal | 18829-55-5 | C7H12O | 112.2 | | 1332.5 | 690.341 | 1.66156 | Dimer |
| 14 | Octanal | 124-13-0 | C8H16O | 128.2 | | 1297.3 | 630.233 | 1.40952 | Monomer |
| 15 | Octanal | 124-13-0 | C8H16O | 128.2 | | 1297.3 | 630.233 | 1.81627 | Dimer |
| 16 | 1-Pentanol | 71-41-0 | C5H12O | 88.1 | | 1259.8 | 556.61 | 1.25322 | Monomer |
| 17 | 1-Pentanol | 71-41-0 | C5H12O | 88.1 | | 1260.5 | 557.925 | 1.51497 | Dimer |
| 18 | Citronellyl formate | 105-85-1 | C11H20O2 | 184.3 | | 1274.2 | 584.219 | 1.96881 |  |
| 19 | 2-Pentylfuran | 3777-69-3 | C9H14O | 138.2 | | 1236.4 | 514.435 | 1.24973 |  |
| 20 | (E)-2-Hexenal | 6728-26-3 | C6H10O | 98.1 | | 1225.3 | 495.575 | 1.17904 | Monomer |
| 21 | (E)-2-Hexenal | 6728-26-3 | C6H10O | 98.1 | | 1225.1 | 495.33 | 1.51062 | Dimer |
| 22 | 3-Methyl-1-butanol | 123-51-3 | C5H12O | 88.1 | | 1214.2 | 477.52 | 1.24378 | Monomer |
| 23 | 3-Methyl-1-butanol | 123-51-3 | C5H12O | 88.1 | | 1213.2 | 475.876 | 1.48724 | Dimer |
| 24 | Heptanal | 111-71-7 | C7H14O | 114.2 | | 1192.1 | 443.269 | 1.34132 | Monomer |
| 25 | Heptan-2-one | 110-43-0 | C7H14O | | 114.2 | 1188.7 | 437.832 | 1.62513 | Dimer |
| 26 | γ-Heptalactone | 105-21-5 | C7H12O2 | | 128.2 | 1190.5 | 440.706 | 1.65556 |  |
| 27 | Heptanal | 111-71-7 | C7H14O | | 114.2 | 1191.5 | 442.38 | 1.68991 | Dimer |
| 28 | Heptan-2-one | 110-43-0 | C7H14O | | 114.2 | 1187.3 | 435.757 | 1.26053 | Monomer |
| 29 | 1-Penten-3-ol | 616-25-1 | C5H10O | | 86.1 | 1167.9 | 406.354 | 0.93811 |  |
| 30 | 1-Butanol | 71-36-3 | C4H10O | | 74.1 | 1153.1 | 385.319 | 1.18027 | Monomer |
| 31 | 1-Butanol | 71-36-3 | C4H10O | | 74.1 | 1152.5 | 384.383 | 1.38472 | Dimer |
| 32 | Diallyl sulfide | 59-28-81 | C6H10S | | 114.2 | 1141.3 | 369.225 | 1.12358 |  |
| 33 | Pentyl acetate | 628-63-7 | C7H14O2 | | 130.2 | 1151 | 382.397 | 1.31673 |  |
| 34 | (E)-2-Pentenal | 1576-87-0 | C5H8O | | 84.1 | 1140.1 | 367.65 | 1.35757 |  |
| 35 | 2-Methyl-1-propanol | 78-83-1 | C4H10O | | 74.1 | 1103.3 | 322.117 | 1.17549 | Monomer |
| 36 | β-pinene | 127-91-3 | C10H16 | | 136.2 | 1095.4 | 313.274 | 1.29237 |  |
| 37 | Dipropyl disulfide | 629-19-6 | C6H14S2 | | 150.3 | 1103 | 321.696 | 1.48121 |  |
| 38 | Hexanal | 66-25-1 | C6H12O | | 100.2 | 1097.2 | 315.135 | 1.56039 |  |
| 39 | 1-Propanol | 71-23-8 | C3H8O | | 60.1 | 1047.7 | 269.553 | 1.11122 | Monomer |
| 40 | 1-Propanol | 71-23-8 | C3H8O | | 60.1 | 1046 | 268.068 | 1.25807 | Dimer |
| 41 | Ethyl 2-methylpropanoate | 97-62-1 | C6H12O2 | | 116.2 | 1003.4 | 234.355 | 1.1948 |  |
| 42 | 2-Furanmethanol acetate | 623-17-6 | C7H8O3 | | 140.1 | 994.9 | 228.285 | 1.42074 |  |
| 43 | Ethanol | 64-17-5 | C2H6O | | 46.1 | 934.5 | 195.149 | 1.14177 |  |
| 44 | Butan-2-one | 78-93-3 | C4H8O | | 72.1 | 910 | 183.076 | 1.24429 |  |
| 45 | Tert-butanol | 75-65-0 | C4H10O | | 74.1 | 921.4 | 188.598 | 1.32775 |  |
| 46 | 4-Hexen-1-ol | 6126-50-7 | C6H12O | | 100.2 | 885.2 | 171.696 | 1.1638 |  |
| 47 | Ethyl pentanoate | 539-82-2 | C7H14O2 | | 130.2 | 897.8 | 177.386 | 1.67653 |  |
| 48 | Sarin | 107-44-8 | C4H10FO2P | | 140.1 | 804.1 | 139.061 | 1.47382 |  |
| 49 | Acrylonitrile | 107-13-1 | C3H3N | 53.1 | | 1007.3 | 237.235 | 1.08503 |  |
| 50 | 2-Pentanone | 107-87-9 | C5H10O | 86.1 | | 991.9 | 226.526 | 1.1203 |  |
| 51 | Methional | 3268-49-3 | C4H8OS | 104.2 | | 904.7 | 180.603 | 1.07498 |  |
| 52 | 3-Hydroxy-2-butanone | 513-86-0 | C4H8O2 | 88.1 | | 1293.2 | 622.69 | 1.0725 |  |
| 53 | 2-Butanol | 78-92-2 | C4H10O | 74.1 | | 1032.4 | 256.839 | 1.3231 | Dimer |
| 54 | 2-Butanol | 78-92-2 | C4H10O | 74.1 | | 1032.8 | 257.168 | 1.15031 | Monomer |
| 55 | 1-Hexanol | 111-27-3 | C6H14O | 102.2 | | 1368.4 | 757.473 | 1.32588 | Monomer |
| 56 | 1-Hexanol | 111-27-3 | C6H14O | 102.2 | | 1368.8 | 758.272 | 1.64272 | Dimer |
| 57 | 2-Acetyl-1-pyrroline | 85213-22-5 | C6H9NO | 111.1 | | 1342.9 | 709.141 | 1.12785 | Monomer |
| 58 | 2-Acetyl-1-pyrroline | 85213-22-5 | C6H9NO | 111.1 | | 1342.6 | 708.599 | 1.47353 | Dimer |
| 59 | Hexyl propionate | 2445-76-3 | C9H18O2 | 158.2 | | 1341.6 | 706.794 | 1.42664 |  |
| 60 | 3-Methyl-2-butenal | 107-86-8 | C5H8O | 84.1 | | 1207.8 | 467.302 | 1.09166 | Monomer |
| 61 | 3-Methyl-2-butenal | 107-86-8 | C5H8O | 84.1 | | 1207.7 | 467.093 | 1.35439 | Dimer |
| 62 | 2-Methyl-1-propanol | 78-83-1 | C4H10O | 74.1 | | 1104.3 | 323.221 | 1.37112 | Dimer |
| 63 | Dimethyl disulphide | 624-92-0 | C2H6S2 | 94.2 | | 1076.1 | 294.751 | 1.14972 |  |
| 64 | 3-Penten-2-one, 4-methyl | 141-79-7 | C6H10O | 98.1 | | 1127.5 | 351.376 | 1.4435 |  |
| 65 | Butanal | 123-72-8 | C4H8O | 72.1 | | 878.5 | 168.724 | 1.11727 | Monomer |
| 66 | Butanal | 123-72-8 | C4H8O | 72.1 | | 880.5 | 169.59 | 1.28126 | Dimer |
| 67 | Diethyl malonate | 105-53-3 | C7H12O4 | 160.2 | | 1076.5 | 295.189 | 1.24811 |  |
| 68 | 2-Furanmethanol, 5-methyl- | 3857-25-8 | C6H8O2 | 112.1 | | 967.4 | 212.528 | 1.25973 |  |
| 69 | (-)-β-Pinene | 18172-67-3 | C10H16 | 136.2 | | 966.6 | 212.09 | 1.30956 |  |

Table S2 Oder description, threshold and ROAV of volatile compounds.

| Number | Volatile compound | Threshold (mg/kg) | Odor description | ROAV^c^ | | | | | | | | | |
| --- | --- | --- | --- | --- | --- | --- | --- | --- | --- | --- | --- | --- | --- |
|  |  |  |  | BB | BLP | BY | HEL | HUL | MG | WN | XJ | XT | ZX |
| 1 | Benzaldehyde | 0.55^a^ | Fruity^b^ | ＜0.1 | ＜0.1 | ＜0.1 | ＜0.1 | ＜0.1 | ＜0.1 | ＜0.1 | ＜0.1 | ＜0.1 | ＜0.1 |
| 2 | (E)-2-Nonenal | 0.00039^a^ | Fatty^b^ | 4.06 | 3.78 | 5.17 | 5.17 | 2.59 | 5.26 | 4.25 | 4.19 | 7.75 | 3.91 |
| 3 | 2-Ethylhexan-1-ol | 0.8^a^ | Citrus^b^ | ＜0.1 | ＜0.1 | ＜0.1 | ＜0.1 | ＜0.1 | ＜0.1 | ＜0.1 | ＜0.1 | ＜0.1 | ＜0.1 |
| 4 | Acetic acid-monomer | 0.0013^a^ | Acidic^b^ | 14.97 | 10.99 | 12.17 | 11.36 | 10.89 | 9.04 | 11.79 | 11.57 | 11.80 | 10.02 |
| 5 | Acetic acid-dimer | 0.0013^a^ | Acidic^b^ | 1.95 | 3.25 | 4.19 | 3.57 | 2.14 | 10.28 | 4.85 | 3.72 | 7.88 | 2.07 |
| 6 | (E)-2-Octenal-monomer | 0.25^a^ | Fatty^b^ | ＜0.1 | ＜0.1 | ＜0.1 | ＜0.1 | ＜0.1 | ＜0.1 | ＜0.1 | ＜0.1 | ＜0.1 | ＜0.1 |
| 7 | (E)-2-Octenal-dimer | 0.25^a^ | Fatty^b^ | ＜0.1 | ＜0.1 | ＜0.1 | ＜0.1 | ＜0.1 | ＜0.1 | ＜0.1 | ＜0.1 | ＜0.1 | ＜0.1 |
| 8 | Nonanal-monomer | 0.0031^a^ | Aldehydic^b^ | 4.19 | 4.37 | 3.89 | 4.40 | 3.13 | 5.22 | 4.06 | 4.09 | 8.11 | 3.42 |
| 9 | Nonanal-dimer | 0.0031^a^ | Aldehydic^b^ | 0.67 | 0.90 | 0.65 | 0.91 | 0.51 | 1.45 | 0.80 | 0.87 | 3.55 | 0.55 |
| 10 | 6-Methylhept-5-en-2-one | 0.3^a^ | Citrus^b^ | ＜0.1 | ＜0.1 | ＜0.1 | ＜0.1 | ＜0.1 | ＜0.1 | ＜0.1 | ＜0.1 | ＜0.1 | ＜0.1 |
| 11 | (E)-2-Heptenal-monomer | 2.4^a^ | Green^b^ | ＜0.1 | ＜0.1 | ＜0.1 | ＜0.1 | ＜0.1 | ＜0.1 | ＜0.1 | ＜0.1 | ＜0.1 | ＜0.1 |
| 12 | (E)-2-Heptenal-dimer | 2.4^a^ | Green^b^ | ＜0.1 | ＜0.1 | ＜0.1 | ＜0.1 | ＜0.1 | ＜0.1 | ＜0.1 | ＜0.1 | ＜0.1 | ＜0.1 |
| 13 | Octanal-monomer | 0.17^a^ | Aldehydic^b^ | ＜0.1 | ＜0.1 | ＜0.1 | ＜0.1 | ＜0.1 | ＜0.1 | ＜0.1 | ＜0.1 | ＜0.1 | ＜0.1 |
| 14 | Octanal-dimer | 0.17^a^ | Aldehydic^b^ | ＜0.1 | ＜0.1 | ＜0.1 | ＜0.1 | ＜0.1 | ＜0.1 | ＜0.1 | ＜0.1 | ＜0.1 | ＜0.1 |
| 15 | 1-Pentanol-monomer | 0.36^a^ | Fermented^b^ | ＜0.1 | ＜0.1 | ＜0.1 | ＜0.1 | ＜0.1 | ＜0.1 | ＜0.1 | ＜0.1 | ＜0.1 | ＜0.1 |
| 16 | 1-Pentanol-dimer | 0.36^a^ | Fermented^b^ | ＜0.1 | ＜0.1 | ＜0.1 | ＜0.1 | ＜0.1 | ＜0.1 | ＜0.1 | ＜0.1 | ＜0.1 | ＜0.1 |
| 17 | 2-Pentylfuran | 0.27^a^ | Fruity^b^ | ＜0.1 | ＜0.1 | ＜0.1 | ＜0.1 | ＜0.1 | ＜0.1 | ＜0.1 | ＜0.1 | ＜0.1 | ＜0.1 |
| 18 | (E)-2-Hexenal-monomer | 0.79^a^ | Green^b^ | ＜0.1 | ＜0.1 | ＜0.1 | ＜0.1 | ＜0.1 | ＜0.1 | ＜0.1 | ＜0.1 | ＜0.1 | ＜0.1 |
| 19 | (E)-2-Hexenal-dimer | 0.79^a^ | Green^b^ | ＜0.1 | ＜0.1 | ＜0.1 | ＜0.1 | ＜0.1 | ＜0.1 | ＜0.1 | ＜0.1 | ＜0.1 | ＜0.1 |
| 20 | 3-Methyl-1-butanol-monomer | 0.0061^a^ | Fermented^b^ | 1.81 | 2.31 | 2.88 | 1.86 | 1.93 | 1.90 | 2.72 | 2.87 | 2.54 | 1.96 |
| 21 | 3-Methyl-1-butanol-dimer | 0.0061^a^ | Fermented^b^ | 0.32 | 0.74 | 0.94 | 0.41 | 0.49 | 0.50 | 1.09 | 1.59 | 0.91 | 0.49 |
| 22 | Heptanal-monomer | 0.26^a^ | Green^b^ | ＜0.1 | ＜0.1 | ＜0.1 | ＜0.1 | ＜0.1 | ＜0.1 | ＜0.1 | ＜0.1 | ＜0.1 | ＜0.1 |
| 23 | Heptan-2-one-dimer | 0.023^a^ | Cheesy^b^ | ＜0.1 | ＜0.1 | ＜0.1 | 0.12 | 0.24 | ＜0.1 | 0.10 | ＜0.1 | 0.33 | ＜0.1 |
| 24 | Heptanal-dimer | 0.26^a^ | Green^b^ | ＜0.1 | ＜0.1 | ＜0.1 | ＜0.1 | ＜0.1 | ＜0.1 | ＜0.1 | ＜0.1 | ＜0.1 | ＜0.1 |
| 25 | Heptan-2-one-monomer | 0.023^a^ | Cheesy^b^ | 0.24 | 0.31 | 0.30 | 0.43 | 0.58 | 0.31 | 0.35 | 0.37 | 0.57 | 0.27 |
| 26 | 1-Penten-3-ol | 4.3^a^ | Green^b^ | ＜0.1 | ＜0.1 | ＜0.1 | ＜0.1 | ＜0.1 | ＜0.1 | ＜0.1 | ＜0.1 | ＜0.1 | ＜0.1 |
| 27 | 1-Butanol-monomer | 0.48^a^ | Fermented^b^ | ＜0.1 | ＜0.1 | ＜0.1 | ＜0.1 | ＜0.1 | ＜0.1 | ＜0.1 | ＜0.1 | ＜0.1 | ＜0.1 |
| 28 | 1-Butanol-dimer | 0.48^a^ | Fermented^b^ | ＜0.1 | ＜0.1 | ＜0.1 | ＜0.1 | ＜0.1 | ＜0.1 | ＜0.1 | ＜0.1 | ＜0.1 | ＜0.1 |
| 29 | Diallyl sulfide | 0.001^a^ | Sulfurous^b^ | 36.37 | 22.49 | 33.26 | 25.41 | 19.76 | 16.59 | 22.32 | 17.69 | 19.82 | 24.77 |
| 30 | Pentyl acetate | 2.45^a^ | Fruity^b^ | <0.1 | ＜0.1 | ＜0.1 | ＜0.1 | ＜0.1 | ＜0.1 | ＜0.1 | ＜0.1 | ＜0.1 | ＜0.1 |
| 31 | (E)-2-Pentenal | 1.4^a^ | Green^b^ | ＜0.1 | ＜0.1 | ＜0.1 | ＜0.1 | ＜0.1 | ＜0.1 | ＜0.1 | ＜0.1 | ＜0.1 | ＜0.1 |
| 32 | 2-Methyl-1-propanol-monomer | 0.033^a^ | Ethereal^b^ | 0.24 | 0.33 | 0.40 | 0.22 | 0.31 | 0.22 | 0.36 | 0.48 | 0.27 | 0.27 |
| 33 | β-pinene | 0.18^a^ | Herbal^b^ | <0.1 | <0.1 | <0.1 | <0.1 | <0.1 | <0.1 | <0.1 | <0.1 | 0.10 | <0.1 |
| 34 | Dipropyl disulfide | 0.13^a^ | Alliaceous^b^ | ＜0.1 | ＜0.1 | ＜0.1 | ＜0.1 | ＜0.1 | ＜0.1 | ＜0.1 | ＜0.1 | ＜0.1 | ＜0.1 |
| 35 | Hexanal | 0.23^a^ | Green^b^ | <0.1 | ＜0.1 | ＜0.1 | ＜0.1 | ＜0.1 | ＜0.1 | ＜0.1 | ＜0.1 | ＜0.1 | ＜0.1 |
| 36 | 1-Propanol-monomer | 0.24^a^ | Alcoholic^b^ | ＜0.1 | ＜0.1 | 0.11 | ＜0.1 | ＜0.1 | ＜0.1 | ＜0.1 | ＜0.1 | ＜0.1 | ＜0.1 |
| 37 | 1-Propanol-dimer | 0.24^a^ | Alcoholic^b^ | ＜0.1 | ＜0.1 | ＜0.1 | ＜0.1 | ＜0.1 | ＜0.1 | ＜0.1 | ＜0.1 | ＜0.1 | ＜0.1 |
| 38 | Ethyl 2-methylpropanoate | 0.00011^a^ | Fruity^b^ | 95.69 | 89.92 | 76.73 | 90.52 | 57.89 | 80.66 | 86.01 | 75.75 | 74.11 | 78.89 |
| 39 | Ethanol | 0.62^a^ | Alcoholic^b^ | ＜0.1 | ＜0.1 | ＜0.1 | ＜0.1 | ＜0.1 | ＜0.1 | ＜0.1 | ＜0.1 | ＜0.1 | ＜0.1 |
| 40 | Butan-2-one | 1.3^a^ | Ethereal^b^ | ＜0.1 | ＜0.1 | ＜0.1 | ＜0.1 | ＜0.1 | ＜0.1 | ＜0.1 | ＜0.1 | ＜0.1 | ＜0.1 |
| 41 | Tert-butanol | 14^a^ |  | <0.1 | ＜0.1 | ＜0.1 | ＜0.1 | ＜0.1 | ＜0.1 | ＜0.1 | ＜0.1 | ＜0.1 | ＜0.1 |
| 42 | Ethyl pentanoate | 0.00058^a^ | Fruity^b^ | 14.87 | 4.81 | 10.08 | 4.59 | 8.21 | 4.63 | 4.24 | 2.99 | 3.14 | 5.91 |
| 43 | Acrylonitrile | 19^a^ |  | ＜0.1 | ＜0.1 | ＜0.1 | ＜0.1 | ＜0.1 | ＜0.1 | ＜0.1 | ＜0.1 | ＜0.1 | ＜0.1 |
| 44 | 2-Pentanone | 0.35^a^ | Fruity^b^ | ＜0.1 | ＜0.1 | ＜0.1 | ＜0.1 | ＜0.1 | ＜0.1 | ＜0.1 | ＜0.1 | ＜0.1 | ＜0.1 |
| 45 | Methional | 0.000063^a^ | Vegetable^b^ | 100.00 | 100.00 | 100.00 | 100.00 | 100.00 | 100.00 | 100.00 | 100.00 | 100.00 | 100.00 |
| 46 | 3-Hydroxy-2-butanone | 0.014^a^ | Buttery^b^ | 0.29 | 0.12 | 0.19 | 0.25 | 0.12 | 0.34 | 0.40 | 0.17 | 0.16 | 0.18 |
| 47 | 2-Butanol-dimer | 0.66^a^ | Fruity^b^ | ＜0.1 | ＜0.1 | ＜0.1 | ＜0.1 | ＜0.1 | ＜0.1 | ＜0.1 | ＜0.1 | ＜0.1 | ＜0.1 |
| 48 | 2-Butanol-monomer | 0.66^a^ | Fruity^b^ | ＜0.1 | ＜0.1 | ＜0.1 | ＜0.1 | ＜0.1 | ＜0.1 | ＜0.1 | ＜0.1 | ＜0.1 | ＜0.1 |
| 49 | 1-Hexanol-monomer | 0.034^a^ | Herbal^b^ | 0.23 | 0.32 | 0.61 | 0.53 | 0.12 | 0.39 | 0.64 | 0.73 | 1.03 | 0.47 |
| 50 | 1-Hexanol-dimer | 0.034^a^ | Herbal^b^ | <0.1 | 0.10 | 0.24 | 0.19 | <0.1 | 0.13 | 0.30 | 0.40 | 0.80 | 0.16 |
| 51 | 2-Acetyl-1-pyrroline-monomer | 0.00003^a^ | Popcorn^b^ | 25.54 | 75.93 | 38.61 | 117.89 | 57.94 | 251.50 | 23.41 | 217.17 | 177.17 | 102.97 |
| 52 | 2-Acetyl-1-pyrroline-dimer | 0.00003^a^ | Popcorn^b^ | 18.15 | 14.36 | 17.44 | 19.40 | 14.06 | 65.30 | 11.37 | 45.31 | 34.96 | 16.82 |
| 53 | 2-Methyl-1-propanol-dimer | 0.033^a^ | Ethereal^b^ | <0.1 | ＜0.1 | ＜0.1 | ＜0.1 | ＜0.1 | ＜0.1 | ＜0.1 | ＜0.1 | ＜0.1 | ＜0.1 |
| 54 | Dimethyl disulphide | 0.0084^a^ | Sulfurous^b^ | 0.33 | 0.14 | 0.11 | 0.13 | 0.52 | 0.10 | 0.15 | ＜0.1 | ＜0.1 | ＜0.1 |
| 55 | 3-Penten-2-one, 4-methyl | 0.07^a^ | Vegetable^b^ | ＜0.1 | ＜0.1 | ＜0.1 | ＜0.1 | ＜0.1 | ＜0.1 | ＜0.1 | ＜0.1 | ＜0.1 | ＜0.1 |
| 56 | Butanal-monomer | 0.1^a^ | Chocolate^b^ | 1.16 | 1.27 | 1.27 | 1.10 | 1.05 | 1.02 | 1.09 | 1.14 | 1.57 | 1.08 |
| 57 | Butanal-dimer | 0.1^a^ | Chocolate^b^ | 0.10 | 0.10 | 0.10 | 0.10 | 0.10 | 0.10 | 0.10 | 0.10 | 0.10 | 0.10 |
| 58 | (-)-β-Pinene | ＞2^a^ | Herbal^b^ | ＜0.1 | ＜0.1 | ＜0.1 | ＜0.1 | ＜0.1 | ＜0.1 | ＜0.1 | ＜0.1 | ＜0.1 | ＜0.1 |

a respects that odor threshold according to Van Gemert (2015).

b respects that odor description according to http://www.thegoodscentscompany.com/search2.html.

c ROAV: relative odor activity value.
